# Sociodemography, Geography, and Personality as Determinants of Car Driving and Use of Public Transportation

**DOI:** 10.3390/bs10060093

**Published:** 2020-05-26

**Authors:** John Magnus Roos, Frances Sprei, Ulrika Holmberg

**Affiliations:** 1 Division of Psychical Resource Theory, Chalmers University of Technology, SE-412 96 Gothenburg, Sweden; Frances.Sprei@chalmers.se; 2 Centre for Consumer Research, School of Business, Economics and Law, University of Gothenburg, SE-405 30 Gothenburg, Sweden; Ulrika.holmberg@cfk.gu.se; 3 School of Health Sciences, Department of Social Psychology, University of Skövde, SE-541 28 Skövde, Sweden; 4 Department of Business Administration and Textile Management, University of Borås, SE-501 90 Borås, Sweden

**Keywords:** public transportation, car use, sociodemography, geography, personality, behavior change

## Abstract

To address the sustainability challenges related to travel behavior, technological innovations will not be enough. Behavioral changes are also called for. The aim of the present study is to examine the influence of sociodemography, geography, and personality on car driving and use of public transportation. Sociodemographic factors have been defined by age, gender, income, and education. Geographic factors have been studied through residential area (e.g., rural and urban areas). Personality has been studied through the Five-Factor-Model of personality—degree of Openness, Conscientiousness, Extraversion, Agreeableness, and Neuroticism. The analysis is based on a survey with 1812 respondents, representative for the Swedish population. Regarding sociodemographic factors, car driving is explained by being male, higher age, higher income, while use of public transportation is explained by lower age and higher education. The user profile of a car driver is the opposite to that of a public transport passenger when it comes to geographic factors; urban residential area explains public transportation while rural area explains car driving. Some personality factors are also opposites; a low degree of Openness and a high degree of Extraversion explain car driving, while a high degree of Openness and a low degree of Extraversion explain use of public transportation. Moreover, car driving is explained by a low degree of Neuroticism, while use of public transportation is explained by a low degree of Conscientiousness and a high degree of Agreeableness. Since sociodemography, geography, and personality influence how people process information and evaluate market propositions (e.g., products and services), the findings presented here are useful for policymakers and transportations planners who would like to change behavior from car driving to public transportation use. Caution should be taken in interpreting the relationship between personality traits and transportation modes, since the personality traits are measured by a short scale (i.e., Big Five Inventory (BFI)-10), with limitations in the factor structure for a representative sample of the Swedish population.

## 1. Introduction

Sweden has set the goal to reduce carbon emissions in the transport sector by 70% from 2010 to 2030 and reach net neutrality by 2045 [1]. In order to achieve this ambitious goal, behavioral changes as well as a shift toward a transport-lean society will be required [1,2,3]. There is a need for policies and interventions that will shift mobility patterns from individual car use to more sustainable transport modes, for instance public transportation. In order to shift toward more sustainable mobility patterns, we need to better understand the users of the different modes.

Although numerous efforts have been made to promote public transportation in many countries, the car continues to be the dominant mode [4]. The reasons are both instrumental, in terms of saving time and effort (e.g., to be able to participate in different activities), and symbolic in terms of status and identity [5,6,7]. Instrumental motives are more common among people in rural areas, where public transportation is less available [8,9]. Symbolic motives are more frequent among males, those less educated, younger people, and those living in rural areas [10]. 

In countries like Sweden where a relatively large part of the population lives in sparsely populated areas, the car is almost indispensable for social and cultural activities. Persons who use the car experience more subjective well-being related to traveling, compared to persons who use public transportation [11]. Therefore, there seems to be a trade-off between individual subjective well-being resulting from traveling by car and the political goal of reducing emissions related to car use. 

According to previous research, the user’s sociodemography and geography influence everyday travel behaviors and mode choices. Car use is positively related to age and income, while use of public transportation is negatively related to age and income. Men also use the car more than women, while women use public transportation more often [9,11,12,13,14,15]. Sociodemographic (such as age, gender, socioeconomic status) and geographic factors (such as residential area) are well-studied in relation to both car use and use of public transportation. However, few studies have investigated the impact of personality (e.g., degree of Extraversion).

Personality gives both consistency and individuality to a person’s behavior [16,17,18]. The Five-Factor-Model of personality (the FFM) is the most popular taxonomy of personality (sometimes refered to as the Big Five), positing that there are five major and universal factors of personality—Openness, Conscientiousness, Extraversion, Agreeableness, and Neuroticism [17,18]. Openness is the degree to which a person is open to new information and would like to try out new things. Conscientiousness is the degree to which a person plans and does a job thoroughly. Extraversion is the degree to which a person is socially outgoing and stimulated from the external environment. Agreeableness is the degree to which a person trusts in others and would like to collaborate with others. Neuroticism is the degree to which a person is anxious and emotionally instable [17]. The FFM model can be translated into other established measurements [16]. For example, materialism could be largely explained in terms of low degree of Openness and Agreeableness, and high degree of Extraversion [19]. Therefore, it can be assumed that people with these traits are using cars more frequently for materalistic (e.g., symbolic) reasons than people with a high degree of Openness and Agreeablness, and a low degree of Extraversion. Environmental preferences could largely be explained in terms of a high degree of Openness and Agreeableness [20,21]. We therefore assume that people with a high degree of such traits will choose public transportation over cars, to protect the environment.

As previously mentioned, few studies have been performed on personality and preferred transport mode. To the best of our knowledge, only one study has explored the relationship between the FFM and choice of transportation mode—a study on the FFM and public transport passengers in Iran [15]. The results from that study show that the use of public transportation is positively related to a high degree of Extraversion and a high degree of Neuroticism. Other studies have looked at other personality frameworks than the FFM. One study, using the Minnesota Personality Inventory (MMPI) [22], found that persons with a high degree of Extraversion take more long-distance car trips [23]. Another study found a positive relation between resistance to change (i.e., low degree of Openness) and car habit [24].

In the present study, we aim to address the identified gap in the literature by exploring personality factors (i.e., the FFM) behind car use and use of public transportation. The study will address the following two research questions:

1. What are the effects of sociodemography, geography, and personality on car use?
2. What are the effects of sociodemography, geography, and personality on use of public transportation?

## 2. Methods

### 2.1. Sample Design and Data Collection 

The data were collected through the SOM-institute (the institute for Society, Opinion, and Media) at the University of Gothenburg, Sweden. The institute collects data from individuals on a wide variety of issues such as political opinion, media use, consumption, occupation, education, health, and well-being. This material is made available for researchers and is approved by the guidelines of the National Committee for Ethics in Sweden. 

In 2017, a mail survey was sent out to 3344 randomly selected Swedish citizens in the 18 to 85 age range. Each participant received the survey by mail in the beginning of September 2017; this was followed up by seven reminders until the end of the year. The respondents were given the opportunity to answer by returning the mail survey or through an electronic survey. For participation, a unique identification code was required that could only be used once. The questions about transportation habits, sociodemography, geography, and personality used in this study were just a small part of the survey [25].

### 2.2. Variables, Measurements and Analysis

The sociodemographic variables of interest were age, gender, income and education. Age was measured with an open-ended question (i.e., 18–85). Gender was measured with three response alternatives—female, male, and others. Respondents that selected the alternative “others” were free to define their gender through an open-ended question. Income was measured by “What is the annual income of all members in your household, before taxes (inluding pension, study allowance etc.)”, dummy coded as 1 (<SEK 300,000), 2 (SEK 300,000–700,000), 3 (>SEK 700,000). Educational attainment was measured by “What is your highest accomplished degree of education?”, categorized as primary school (coded as 1), high-school (coded as 2), bachelor degree or above (coded as 3). 

The geographic variable of interest was the residential area, which was measured with four alterantives—rural area (coded as 1), small town (coded as 2), medium-sized town/city (coded as 3), large city (i.e., Stockholm, Gothenburg or Malmö) (coded as 4). 

Personality was assessed using the Big Five Inventory, BFI-10 [26], which is a 10-item inventory with 2 items measuring each personality factor. The introduction text to the ten items was, “To what degree do the following statements correspond to who you are”, “I view myself as someone who …”. Personality factor items were measured using a four-point Likert scale ranging from 1 (“strongly disagree”) to 4 (“strongly agree”). The Openness factor was constructed by averaging the responses to “…has few artistic interests” (reversed) and “…has an active imagination”. The Conscientiousness factor was constructed by averaging the responses to “…tends to be lazy” (reversed) and “…does a thorough job”. The Extraversion factor was constructed by averaging the responses to “…is reserved” (reversed) and “…is outgoing, sociable”. The Agreeableness factor was constructed by averaging the responses to “…tends to find fault with others” (reversed) and “…is generally trusting”. The Neuroticism factor was constructed by averaging the responses to “…is relaxed, handles stress well” (reversed) and “…gets nervous easily” [26]. Only respondents who had provided answers on both items to a specific factor were included in the analyses. 

The internal consistency of personality factors was tested through both interitem correlations (*r*) and Cronbach’s alpha coefficients (*α*), and the FFM factor structure was tested through an exploratory factor analysis (maximum likelihood as extraction method; Varimax with Kaiser Normalization as rotation method).

The internal consistency and descriptive statistics of the personality factors measured by the BFI-10 were—Openness (*α* = 0.18, *r* = 0.10, *p* < 0.01, *M* = 2,56, *SD* = 0.74, *N* = 1715) , Conscientiousness (*α* = 0.36, *r* = 0.22 *p* < 0.01, *M* = 3.27, *SD* = 0.57, *N* = 1715), Extraversion (*α* = 0.63, *r* = 0.46, *p* < 0.01, *M* = 2.84, *SD* = 0.72, *N* = 1708), Agreeableness (*α* = 0.06, *r* = 0.03, *M* = 3.06, *SD* = 0.52, *N* = 1705), Neuroticism (*α* = 0.62, *r* = 0.45, *p* < 0.01, *M* = 2.20, *SD* = 0.70, *N* = 1715). 

The assumed FFM factor structure could not be replicated in a Swedish context, neither in the present study nor in a previous study with the same sampling procedure as in the present study [27]. In fact, only Extraversion and Neuroticism were factors with high discriminant validity [27]. Despite limitations in the internal consistency of specific factors and in the overall factor structure, a comparative analysis of short-scales ranked the BFI-10 as the most reliable and valid short scale of the FFM in a Swedish context [28]. The two items for each factor are selected to represent the broadness (i.e., all facets) of the specific factor. Compared to other short-scales, the BFI-10 therefore has a high content validity [26,28]. Short scales have the advantage that they decrease the response time and can thus be included in larger surveys (as the one used in this study). Response time also has a positive impact on the response rate and representativeness of the survey.

Car driving was measured with “How often have you driven a car during the last twelve months?”. Use of public transportation was measured with “How often have you used public transportation during the last twelve months?”. To each of the two questions, the respondents were asked to indicate their frequencies on a seven-point scale—never (coded as 1), occasionally during the past 12 months (coded as 2), occasionally every 6 months (coded as 3), occasionally every 3 months (coded as 4), occasionally every month (coded as 5), occasionally every week (coded as 6), several times a week (coded as 7). 

IBM SPSS Statistics 25 was used to obtain descriptive statistics of sample characteristics. For each transportation mode (i.e., car driving and public transportation, respectively), an ordinal regression analysis was applied to test the effects of sociodemography, geography, and personality, using IBM SPSS Statistics 25. The choice of regression model was based on the ordinal nature of the dependent variables. In ordinal regression, only the relative order between response alternatives is of interest and the ordinality of the responses are considered through a series of comparison models [29]. 

## 3. Results

### 3.1. Characteristics of the Sample 

The response rate was 53% (*N* = 1785). In total, 200 respondents (11.2%) used the electronic survey instead of the mail survey. Among the 1785 respondents, 836 (47%) were male, 945 (53%) were female and 4 were “others”. Since the group “others” was so small it was excluded from the gender analyses. The mean age (*N* = 1785) was 52.3 years. The annual household income (*N* = 1645) was distributed as follows—24% < SEK 300,000; 45% SEK 300,000–700,000; 31% > SEK 700,000. The highest educational attainment (*N* = 1716) had the following distribution—16% primary school; 53% high school; 31% bachelor degree or above. The distribution of the residential area (*N* = 1733) was: 15% rural areas; 17% smaller towns; 51% middle-sized towns/cities; 17% large cities (i.e., Stockholm, Gothenburg or Malmö). 

Regarding car driving (*N* = 1756), 15% of the respondents reported that they had not driven a car at all during the last 12 months; 5% reported that they drove a car less than a few times every month (alternative 2–4 on the ordinal scale); 5% reported that they drove a car a few times every month; 12% reported that they drove a car a few times every week; 63% reported that they drove a car several times a week.

Regarding use of public transportation (*N* = 1739), 23% of the respondents reported that they had not used public transportation at all during the last 12 months; 33% reported that they used public transportation less than a few times every month (i.e., 11% a few times a year, 9% a few times every 6 months, 13% few times every 3 months); 16% had used public transportation a few times every month; 8% had used public transportation a few times every week; 20% used public transportation several times a week.

### 3.2. The Effects of Sociodemography, Geography, and Personality on Car Driving 

An ordinal regression analysis was conducted with frequency of car driving as the dependent variable, and sociodemography, geography, and personality as independent variables. The results are presented in Table 1. From the sociodemographic variables, we find that car driving is predicted by being male, higher age, and income. Car driving is also more frequent in rural areas. Residential area is the variable with the strongest predictive value. Regarding the Big Five personality factors, we find that Openness, Extraversion, and Neuroticism are significant at the 5% level (Table 1). Openness and Neuroticism both have negative coefficients, implying that more frequent car drivers have lower degrees of these traits than less frequent car drivers. Extraversion has positive coefficients, implying that more frequent car drivers have higher degrees of this trait compared to less frequent car drivers. 

### 3.3. The Effects of Sociodemography, Geography, and Personality on Use of Public Transportation 

As with driving, an ordinal regression analysis was conducted with frequency of public transportation as dependent variable, and sociodemography, geography, and psychography as independent variables. We present the results in Table 2. 

Interestingly, the independent variables explaining use of public transportation are almost the opposite of the independent variables predicting frequency of car use. A comparative analysis of Table 1 and Table 2 indicates that users of the two transportations modes differ a lot with respect to sociodemography, geography and personality. Again, residential area has the highest explanatory value, but in this case with the opposite sign of car driving, i.e., the more urban the area of residence, the higher the frequency of public transportation use. Residential area is followed by age, again with a negative coefficient, implying that younger people use public transportation more frequently. Higher education also implies more frequent use of public transportation. As in the case of driving, the influence of personality factors is lower than that of the geographic factor, but still significant at the 5% level for four out of five factors. Openness and Agreeableness are both positively correlated with use of public transportation, while Conscientiousness and Extraversion have negative coefficients. Neuroticism does not have any predictive value on use of public transportation. 

## 4. Discussion 

### 4.1. What Are the Effects of Sociodemography, Geography, and Personality on Car Use? 

Our results regarding demography are supported by previous research. Car use is positively related to being male and older age [9,11,12,13,14]. We also find that car use is positively related to income, but not education. An explanation for this result could be that people with a higher education are more informed about the negative environmental impact of car driving, which might have the opposite effect on car driving compared to being financially able to drive. There could also be structural explanations, such as people with higher education living in more central areas and/or having more flexible jobs allowing them to be less car-dependent. We suggest further research in order to better understand why income better predicts car driving than educational attainment and therefore behavioral restrictions in car use. 

Regarding geography, our results correspond to previous studies [8,9]. Car use is more common in rural areas. The scale of residential area used in the present study is very coarse and thus does not allow for more advanced analysis of the relationship between residential area and car driving frequencies (see e.g., [30,31]). However, one reason for higher driving frequencies in rural areas compared to urban areas, is simply that public transportation is less available and thus car dependency is stronger. 

The most novel contribution of the present study is related to the FFM, since many of the findings have not been previously reported. The relation between car driving and low degree of Openness might be explained by several factors related to the FFM, such as close-mindedness to new information and resistance to behavioral change [16,17]. It might also be explained by materialistic and symbolic preferences of car use among people with a low degree of Openness [19]. A third explanation might be that people with a low degree of Openness tend to be less concerned about the environment [20,21,32] and therefore care less about the negative environmental effects of car use. Our results correspond to a previous study that associated car habit with resistance to change [24]. 

People with a high degree of Extraversion use the car more frequently, probably because they are engaged in more activities outside their household and live more active lives, compared to their more introvert counterparts [16,17]. This conclusion is supported by previous research, that shows that Extraversion is positively related to non-work-related car use [23]. A high degree of Extraversion as a predictor of car use might also be explained by materialistic and symbolic motives [19]. 

The negative influence of Neuroticism on car use might be explained by a perceived stress related to driving cars, and people stating a high degree of Neuroticism might therefore avoid traveling by car. 

We did not find any significant relation between car use and Conscientiousness, which might be explained by several conflicting values of car use among people with high Conscientiousness, for instance self-control in everyday life versus long-term environmental effects [16,17]. Further, we did not find that people with a high degree of Agreeableness drive a car less frequently, which we expected from their nonmaterialistic and pro-environmental character [19,20,21,32]. Many of these issues should be further studied for a more in depth understanding of the personality behind frequent car use, in order to facilitate behavioral change.

### 4.2. What Are the Effects of Sociodemography, Geography, and Personality on Use of Public Transportation? 

Regarding sociodemography and use of public transportation, our findings correspond to previous research. Use of public transportation is positively related to education, and negatively related to age [9,12,15]. Our results regarding geography and use of public transportation match previous research. Use of public transportation is more common in urban areas where public transportation is more available [9].

The positive correlation between frequent public transportation and a high degree of Openness might be explained by several factors. People who score high on Openness are more susceptible to environmental information and more willing to try alternatives. Generally, they are more open to new information and more willing to change their behavior [16,17,18]. It might also be explained by environmental concerns and behaviors [20,21,32], and less materialistic and symbolic motives for car use [19]. 

A low degree of Conscientiousness among people who frequently use public transportation might be explained by their lower need for self-control [16,17]. The correlation between a low degree of Extraversion and use of public transportation is difficult to explain and is also contradictory to some previous findings [15]. It could be expected that people who are more outgoing and social also ride together on public transportation, rather than driving cars. The contradictory findings might be related to cultural differences; if traveling with public transportation is perceived as an opportunity for social interaction or not. One explanation could be that people with high Extraversion have more activities outside their homes and public transportation cannot meet the transportation needs related to these.

People with a high level of Agreeableness travel more often by public transportation, which can be expected given their ambition to reach agreements [16,17,18], their environmental concern [32], and their lower values of materialism [19]. 

Finally, in contrast to previous research [15], we did not find a negative relation between Neuroticism and use of public transportation. This difference might be related to different cultures in Iran and Sweden, and different perspectives and experiences of public transportation. More research from various countries and cultures is needed, in order to further explore the relationship between Neuroticism and use of public transportation, as well as a further understanding of the relationship between the FFM and the use of public transportation and car driving.

### 4.3. Limitations 

The results of this study must be viewed in the light of its limitations. First, the participants’ transport behaviors were self-reported, which causes well-known problems in the field of social and personality psychology [33]. We recommend future researchers pay more attention to measuring car use and use of public transportation from real behavior, for instance through GPS tracking in smartphone applications or other automatic behavioral registration techniques (e.g., [34]).

Second, we only measured frequency of travel and not distance traveled.

Third, although the ambition was to provide a representative sample of the Swedish population (within the 18–85 age range), the sample slightly under-represents young adults (18–29), males, urban citizens, and people with non-Swedish citizenship [25]. Comparison of the composition of our sample with the Swedish population [35] revealed that our sample has a higher mean age and is somewhat over-represented by female citizens. In the group 18–85 years in 2017 (the year of data collection), the mean age in the population was 48.1 compared to the mean age of our respondents of 52.3. In 2017, 50% of the population in the age-group 18-85 were female and 50% were male, whereas 53% of the respondents in our sample were female and 47% were male [25,35].

Fourth, the FFM was measured by a short scale (i.e., BFI-10). The advantages of a short scale have been discussed in the Methods section. However, a short scale has substantial losses and psychometric disadvantages compared to a full-length scale [26,28,36]. As discussed in the Methods section, the empirical data presented here could neither confirm the internal consistency of Agreeableness nor the discriminant validity of three factors—Openness, Conscientiousness, and Agreeableness. The factor structure of BFI-10 in the present study finds the same structure as that in a previous study of a Swedish nationally representative sample [27]. 

Fifth, caution should be taken in generalizing the present results outside of Sweden. Even though the FFM mostly has shown universal characteristics, transport behavior might depend on different factors in different cultures. We have for instance seen that personality traits influence public transportation differently in Sweden and Iran. When it comes to sociodemographic and geographic factors, the results of the present study correspond to previous findings. At the same time, we know that transport choices are related to the infrastructural network of society and/or cultural values. In Singapore for instance, public transportation is used for grocery shopping to a much larger extent than in most other countries, Sweden included [37]. 

Finally, we would like to comment on the overall ability to predict mode choices with the two models presented in this paper (Table 1 and Table 2). The variability in car use explained by the first model is 25.5% (Nagelkerke R²). The variability in use of public transportations explained by the second model is 29.4% (Nagelkerke R²). Although comparable to other models in this field [38,39,40,41], 74.5 and 70.6% of the variability of the independent variables remain unexplained. To achieve a higher explanatory value, more detailed information is needed on, e.g., residential location, distance to work, type of occupation, number of children. The effect of residential area has been studied more extensively in previous papers and characteristics of the area of residence influencing mode choice is well-known [30]. This is confirmed in our study, since it is the best predictor and thus, omitting it would make it a confounding factor. The main purpose and contribution of the present study was however to look beyond obvious and well-known factors, and instead focus on latent psychological factors. Since the FFM model of personality seems to have quite modest influence on mode choices, we recommend a careful interpretation of the results and further research on the relationship between the FFM and mode choice.

## 5. Conclusion and Implementations

Several sociodemographic, geographic, and personality factors influence the frequency of car use and public transportation use. High income might indicate economic ability to drive a car. Living in rural areas, with less accessibility to public transportation, might imply more car dependency [8,9]. Furthermore, positive symbolic values related to cars are stronger in rural areas [11]. Therefore, it seems as if both instrumental and symbolic motives can contribute to the strong geographic effect on car use and public transportation. A behavioral change toward public transportation in rural areas probably needs to combine better accessibility to public transportation with less positive symbolic values related to car driving. 

As discussed in the introduction, a high degree of Extraversion and a low degree of Openness and Agreeableness can be translated into materialistic traits [19]. Further, environmental preferences can largely be explained in terms of a high degree of Openness and Agreeableness [20,21]. The present study indicates that car driving is positively related to a low degree of Openness and a high degree of Extraversion, while use of public transportation is related to a high degree of Openness, a low degree of Extraversion, and a high degree of Agreeableness. Based on the findings of the present study, it is appealing to conclude that car driving is associated with materialism and that use of public transportation is associated with environmental preferences. However, we need more advanced analyses to explore these relationships, for instance through structural equation modelling. It might be that materialism and environmental preferences are better predictors of car driving and use of public transportations than the FFM. 

If the challenge is to reduce car use in society, this study’s results might be useful for psychologists, policymakers, and transportation planners who would like to change travel behavior from car use to public transportation. Our results show that frequent car drivers are characterized by older age, being men, having higher income, rural living, low Openness, high Extraversion, and low Neuroticism. In order to facilitate a behavioral change among these people, we need to learn more what motivates them, because no single design suits everyone [42]. The results presented here can be useful in infrastructural design, service design, and communication schemes to targeted groups. We know that people, depending on their sociodemography, geography, and personality, are attracted by different kinds of design and communication appeals [42,43,44].

We find a negative correlation between Extroversion and use of public transportation. In order to shift people with a high degree of Extraversion from car use to public transportation, more investments in internal design and more enjoyable public transport might be needed. The reason is that people with high Extraversion are especially stimulated from their external environment. Reduced congestion on public transportation vehicles could also attract extrovert travelers, since overcrowded vehicles reduce the possibility of viable social interactions. An alternative approach might be to make car use less attractive for people with high Extraversion, for instance by reducing the pleasure element of car use and making urban environments less accessible by car.

Shifting social norms, meanings, and values might also influence people with high Extraversion. This might imply an increased emphasis on communicating negative attributes of car use, such as selfishness and negative environmental impact. Values related to public transportation are probably already appealing for people high on Agreeableness and Openness, but in order to attract more extroverted people, public transport needs to be the social norm and be associated with a higher status for the users. 

In this study we use a short scale, the BFI-10, which comes with its advantages and disadvantages. A shorter scale has a positive effect on response rate and representativeness but has limitations when it comes to factor structure and internal consistency. Given the limitations, the results should be interpreted with caution [28]. Still, our results show that there are relationships between personality and mode choice that merit further studies through, e.g., more reliable scales to measure FFM. We also recommend researchers to go beyond the established FFM and look at other personality factors that are not included in the FFM, but still exist in the general population, such as borderline personality traits [45].

## Figures and Tables

**Table 1:** Ordinal regression analysis to predict car use.

Predictors	Estimate	*SE*	*P* Value	*OR*	95% *CI*
Sociodemography					
Age (in years)	0.02	0.00	**<0.001**	1.02	[0.01, 0.02]
Gender (RG: Male)	−0.83	0.12	**<0.001**	0.44	[−1.06, −0.59]
Income (RG: High income)					
Medium income	−0.37	0.14	**=0.009**	0.69	[−0.65, −0.09]
Low income	−1.39	0.17	**<0.001**	0.25	[−1.72, −1.07]
Education (RG: High education)					
Medium education	0.18	0.13	=0.157	1.20	[−0.07, 0.44]
Low education	−0.36	0.20	=0.079	0.70	[−0.75, 0.04]
Geography (Residential area)					
(RG: Large city)					
Medium-sized town/city	0.89	0.14	**<0.001**	2.44	[0.61, 1.17]
Small town	1.63	0.20	**<0.001**	5.10	[1.24, 2.01]
Rural area	2.86	0.27	**<0.001**	17.46	[2.32, 3.39]
Personality					
Openness	−0.17	0.08	**=0.028**	0.84	[−0.33, −0.02]
Conscientiousness	0.17	0.10	=0.102	1.19	[−0.03, 0.37]
Extraversion	0.23	0.08	**=0.006**	1.26	[0.07, 0.40]
Agreeableness	−0.16	0.11	=0.158	0.85	[−0.38, 0.06]
Neuroticism	−0.20	0.09	**=0.021**	0.82	[−0.37, −0.03]

*Note.* Bold value entries imply statistically significant effects (*p* ˂ 0.05). *SE* = stander error; *OR* = odds ratio; *CI* = confidence interval of the estimates. The question was “How often have you driven a car during the past twelve months?” and the response scale was a seven-point ordinal scale ranging from 1 (“never”) to 7 (“several days a week”). Scales for independent variables were as follows—age 18–85; gender, 0 or 1; educational attainment 1–4 (ordinal scale); household income, 1–3 (ordinal scale); residential area scale 1–4 (ordinal scale); personality factors 1–4 (ordinal scale, from low degree to high degree). RG = Reference Group.

**Table 2:** Ordinal regression analysis to predict use of public transportation.

Predictors	Estimate	*SE*	*P* Value	*OR*	95% *CI*
Sociodemography					
Age (in years)	−0.03	0.00	**<0.001**	0.97	[−0.03, −0.02]
Gender (RG: Male)	0.19	0.10	=0.060	1.21	[0.01, 0.38]
Income (RG: High income)					
Medium income	−0.18	0.11	=0.100	0.84	[−0.40, 0.04]
Low income	0.22	0.14	=0.124	1.25	[−0.06, 0.50]
Education (RG: High education)					
Medium education	−0.58	0.11	**<0.001**	0.56	[−0.80, −0.37]
Low education	−0.95	0.17	**<0.001**	0.39	[−1.28, −0.61]
Geography (Residential area)					
(RG: Large city)					
Medium-sized town/city	−1.35	0.14	**<0.001**	0.26	[−1.62, −1.07]
Small town	−2.09	0.17	**<0.001**	0.12	[−2.43, −1.76]
Rural area	−2.55	0.18	**<0.001**	0.08	[−2.91, −2.19]
Personality					
Openness	0.20	0.07	**=0.003**	1.22	[0.07, 0.32]
Conscientiousness	−0.17	0.09	**=0.049**	0.84	[−0.34, 0.00]
Extraversion	−0.14	0.07	**=0.048**	0.87	[−0.28, 0.00]
Agreeableness	0.26	0.10	**=0.005**	1.30	[−0.08, 0.45]
Neuroticism	−0.04	0.08	=0.581	0.96	[−0.19, 0.11]

*Note.* Bold value entries imply statistically significant effects (*p* ˂ 0.05). *SE* = stander error; *OR* = odds ration; *CI* = confidence interval of the estimates. The question was “How often have you used public transportation during the past twelve months?” and the response scale was a seven-point ordinal scale ranging from 1 (“never”) to 7 (“several days a week”). Scales for independent variables were as follows—age 18–85; gender, 0 or 1; educational attainment 1–4 (ordinal scale); household income, 1−3 (ordinal scale); residential area scale 1–4 (ordinal scale); personality factors 1–4 (from low degree to high degree). RG = Reference Group.
